# Pollution Characteristics of Heavy Metals in PM_1_ and Source-Specific Health Risks in the Tianjin Airport Community, China

**DOI:** 10.3390/toxics12080601

**Published:** 2024-08-18

**Authors:** Jingbo Zhao, Jingcheng Xu, Yanhong Xu, Yaqin Ji

**Affiliations:** 1 College of Transportation Science and Engineering, Civil Aviation University of China, Tianjin 300300, China; jbzhao@cauc.edu.cn (J.Z.);; 2 College of Environmental Science and Engineering, Nankai University, Tianjin 300350, China

**Keywords:** PM_1_, heavy metals, positive matrix factorization, source-specific health risk, airport-related pollution

## Abstract

The airport and its surrounding areas are home to a variety of pollution sources, and air pollution is a recognized health concern for local populated regions. Submicron particulate matter (PM_1_ with an aerodynamic diameter of <1 mm) is a typical pollutant at airports, and the enrichment of heavy metals (HMs) in PM_1_ poses a great threat to human health. To comprehensively assess the source-specific health effects of PM_1_-bound HMs in an airport community, PM_1_ filter samples were collected around the Tianjin Binhai International Airport for 12 h during the daytime and nighttime, both in the spring and summer, and 10 selected HMs (V, Cr, Mn, Co, Ni, Cu, Zn, As, Cd, and Pb) were analyzed. The indicatory elements of aircraft emissions were certified as Zn and Pb, which accounted for more than 60% of the sum concentration of detected HMs. The health risks assessment showed that the total non-cancer risks (TNCRs) of PM_1_-bound HMs were 0.28 in the spring and 0.23 in the summer, which are lower than the safety level determined by the USEPA, and the total cancer risk (TCR) was 2.37 × 10^−5^ in the spring and 2.42 × 10^−5^ in the summer, implying that there were non-negligible cancer risks in the Tianjin Airport Community. After source apportionment with EF values and PMF model, four factors have been determined in both seasons. Consequently, the source-specific health risks were also evaluated by combining the PMF model with the health risk assessment model. For non-cancer risk, industrial sources containing high concentrations of Mn were the top contributors in both spring (50.4%) and summer (44.2%), while coal combustion with high loads of As and Cd posed the highest cancer risk in both seasons. From the perspective of health risk management, targeted management and control strategies should be adopted for industrial emissions and coal combustion in the Tianjin Airport Community.

## 1. Introduction

With the development of economic globalization and regional economic cooperation, the airport economic zone provides a key factor in the development of national economics. Many industries, logistics, and enterprises are located in the airport economic zone, gradually forming local populated regions. However, the emissions produced by airport activities influence local air quality at and around airports. Adverse health effects are suspected in people working in airports or living near them. Barrett et al. [1] estimated that about 10,000 people worldwide die prematurely each year due to pollution caused by aircraft activities.

The emission of particulate matter (PM), as one of the most important pollutants emitted from aircraft engine exhaust at the airport, have been regulated by the International Civil Aviation Organization (ICAO) in recent years [2]. Merzenich et al. [3] found that the main problem of occupational health hazards facing airport ramp workers due to air pollution was related to exposure to ultra-fine particles from aircraft. The health effect induced by PM highly depends on its characteristics such as particle size: smaller particle sizes were associated with higher toxicity [4,5]. Compared with coarse and fine particulate matter, submicron particulate matter (PM1 with an aerodynamic diameter of <1 mm) has a higher toxic potential [6].

Heavy metals (HMs), which can pose crucial risks to human health, mainly exist in particulate matter in airports ambient environments [7,8,9]. For instance, previous studies have shown that Cr, Co, Ni, As, and Cd are carcinogenic and associated with kidney disease, stroke, and asthma [10]; Zn and Mn can cause cardiovascular and neurological diseases [11]. Since there are various HM sources in the airport area, including aircraft engines, vehicles, and industry, it is crucial to evaluate the contribution of particulate HMs. Conventionally, some studies have detected HMs from aircraft exhausts under controlled in-flight conditions to determine their compositions [12,13,14] and mainly focused on the impact of flight activities on the HM concentrations or variations in the airport area [15,16,17]. Nevertheless, very few studies quantitatively identified possible sources of PM-bound HMs in the airport community [18].

The typical health risk assessment model is commonly utilized to assess the non-cancer and cancer risks of PM-bound HMs [19]. To evaluate the health risks of HMs from diverse sources, some studies quantitatively apportioned health risks to sources by linking the source profiles resolved using receptor models with the health risk assessment model [20,21]. Thus, considering public health protection, focusing on the source-specific health risk instead of the sources’ contributions to the concentrations of PM-bound HMs could be a more effective mitigation strategy for these emissions. 

Given the above, PM_1_-bound HMs (V, Cr, Mn, Co, Ni, Cu, Zn, As, Cd, and Pb) were measured in the Tianjin Binhai International Airport Community by collecting PM_1_ samples during the spring and summer in 2021. The health-risk contributions of diverse sources in the Tianjin Airport Community were assessed with the PMF model in this study, aiming to propose a control strategy with sources based on a quantitative evaluation of the health risks. The specific objectives of this study were to (1) determine the seasonal and diurnal variations in the concentrations of PM_1_-bound HMs; (2) determine the impact of aviation activities on HM concentrations and indicators of aircraft emissions; (3) to quantitatively apportion each source of HM in the Tianjin Airport Community using the PMF model; (4) to assess the health risks from exposure through the inhalation of HMs in PM_1_; and (5) to evaluate the health risks of specific sources by combining the PMF and HR models.

## 2. Sampling and Methodology

### 2.1. Description of the Study Area and Sampling of PM_1_

Tianjin is a municipality located in the Beijing-Tianjin-Hebei metropolitan area in China with an area of 12,000 km^2^, and it has a temperate monsoon climate [22]. As a result of the rapid progress of industrialization and urbanization in recent years, air pollution has become one of the most serious environmental problems in Tianjin. The Tianjin Binhai International Airport is located in the Dongli District of Tianjin at 39°07′ N, 117°20′ E. Tianjin Airport has been listed as a 4E grade airport, and it is also one of the airports with the highest grades in China, with an annual airport passenger throughput of 23 million by the end of 2020.

**Figure 1:**
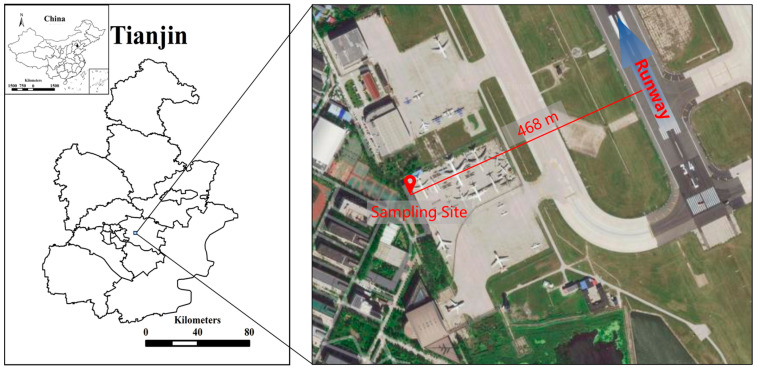
The study area and the PM_1_ sampling site in the Tianjin Binhai Airport Community.

In this study, PM_1_ sampling campaigns were conducted on the roof of a building nearly 4 m above ground on a landing field, 468 m away from the 34 L runway on the southwest side of Tianjin Airport. The sampling site was the only residential area within four kilometers of the airport (Figure 1). The Tianjin Airport Community is surrounded by transportation routes, such as the Expressway and the National Highway. In addition, it is worth to mentioning that there are several trucks and buses with diesel engines over these main traffic routes. The Xinli Industrial Park and Baoyuan Industrial Park are two large-scale industrial parks in Dongli District that are located in the area about 3 and 6 km away from the airport, and they can be seen as the main industrial sources near the sampling site.

The PM_1_ samples were collected on 90 mm Pallflex quartz filters (2500QAT-UP, Pall Life Sciences, Ann Arbor, New York, USA) using a medium-volume air sampler (HY-100SFB, Hengyuan Corporation, QingDao, China) with a flow rate of 100 L·min^−1^. The samples were collected for 12 h during the daytime (from 8:00 A.M. to 8:00 P.M.) and nighttime (from 8:00 P.M. to 8:00 A.M. the next day). A total of 28 samples including 14 daily samples and 14 nightly samples were collected in the spring (from 10 May to 16 May 2021) and 36 samples including 18 daily samples and 18 nightly samples were collected in the summer (9 days from 26 July to 4 August 2021), except for 26 July 2021 because the weather was stormy on that day. 

### 2.2. Chemical Analysis and Quality Control

During our sampling period, 3 blank samples were also collected in each season for contrast in the chemical analysis (unexposed filters stored in foil bags before and after sampling until the time of analysis and processed simultaneously with the field samples). All filters were equilibrated under constant temperature (22 °C ± 1 °C) and relative humidity (30 ± 5%) for 72 h before any gravimetric measurements were performed using an ultra-micro balance. After sampling, all samples were stored and sealed in pre-cleaned plastic cases at −20 °C until analysis. A microwave digestion system and an inductively coupled plasma mass spectrometer (ICP-MS, Agilent 7500a, Agilent Technologies, Santa Clara CA, USA) were employed to analyze a variety of selected HMs in the Teflon-filter PM_1_ samples. The details regarding the extraction and analyzation of the chemical compositions and quality control are described in the SI (Text S1).

### 2.3. Statistical Analysis and Source Apportionment

In this study, Origin 18.0 and Excel were used for statistical analysis. Enrichment factors (EFs) and positive matrix factorization (PMF) were applied to explore the interrelatedness between PM_1_-bound HMs and their possible sources.

#### 2.3.1. Enrichment Factors

The enrichment factor (EF) of heavy element i (${EF}_{i}$) was calculated as the following equation [23]:

(1) $${EF}_{i}=\frac{{\left(C_{i}/C_{ref}\right)}_{atmosphere}}{{\left(C_{i}/C_{ref}\right)}_{crust}}$$

where ${\left(C_{i}/C_{ref}\right)}_{atmosphere}$ is the ratio of the selected element $C_{i}$ and the reference element $C_{ref}$ concentrations (mg/kg) in aerosol samples; ${\left(C_{i}/C_{ref}\right)}_{crust}$ is the ratio of their concentrations in the upper continental crust. 

In general, the reference elements commonly used are Fe, Al, Mn, Sc, and Ti, which were chosen because of their high concentration in the Earth’s crust, stability during the chemical analysis process, and relatively lesser contribution from other sources [24]. In this study, the ${EF}_{i}$ was normalized by the reference element Mn, whose concentration is 583 mg/kg in the upper continental crust in China [25], and Mn has been applied successfully for the data analysis of particulate matter in several studies before [23,26].

Operationally, the value of EF < 10 indicates that the target element is barely affected by human activities; 10 < EF < 100 indicates that the target elements are moderately enriched and come from anthropogenic sources other than the crust. In addition, a value of EF > 100 indicates that the elements are extremely enriched, suggesting that anthropogenic sources are dominant [27].

#### 2.3.2. Positive Matrix Factorization Model

The contribution of HM sources was evaluated by the PMF analysis method (USEPA PMF 5.0). Due to the advantage that PMF can ascertain the contributions of sample sources according to their compositions without any emission inventory, it has been one of the most representative receptor models in source apportionment of pollutants [19,28,29].

The iterative least squares method of PMF is to minimize the objective function (Q) by continuously decomposing the matrix of concentration of HMs (X) into the source profiles matrix (F), source contribution matrix (G), and residual matrix (E) [30]. The principles of the PMF model can be expressed as Equations (2) and (3).

(2) $$X_{ij}=\sum_{k=1}^{p}G_{ik}F_{kj}+E_{ij}$$

(3) $$Q=\sum_{i=1}^{n}\sum_{j=1}^{m}{\left(\frac{X_{ij}-\sum_{k=1}^{p}G_{ik}F_{kj}}{u_{ij}}\right)}^{2}$$

where i and j are the quantity of samples and elements; k is the number of sources, respectively; $X_{ij}$ is the matrix containing i samples and j elements; $G_{ik}$ refer to source k and sample i in the contribution matrix; $F_{kj}$ is the kth source profile of element j; and $E_{ij}$ is the residual. The $u_{ij}$ corresponds to the uncertainty for jth element in the ith sample, which is calculated according to Equations (4) and (5):

(4) $$\mathrm{For}\;X_{ij}\leq MDL,\;u_{ij}=5/6\;\times \;MDL$$

(5) $$\mathrm{When}\;X_{ij}>MDL,\;u_{ij}=\sqrt{{\left(\sigma_{j}\;\times \;X_{ij}\right)}^{2}+{\left(0.5\;\times \;MDL\right)}^{2}}$$

where the *MDL* is the method detection limit. $\sigma_{j}$ is error fraction determined by the measurement uncertainty [31].

### 2.4. Source-Specific Health Risk Assessment

#### 2.4.1. Health Risk Assessment Model

Inhalation exposure is the primary route of direct exposure to atmospheric HMs [32]. Thus, the human health risks of PM_1_-bound HMs were generally calculated using the health risk assessment model. The normal average daily exposure dose (ADD, in mg/kg/d), which estimates the inhalation exposures, can be calculated as follows:

(6) $${ADD}_{i}=\frac{C_{i}\;\times \;InhR\;\times \;EF\;\times \;ED}{BW\;\times \;AT}$$

where $C_{i}$ is the concentration of heavy metal i (mg·m^−3^), InhR represents the inhalation rate (mg·(kg·day)^−1^), EF refers to the exposure frequency (day·a^−1^), ED is the duration of inhalation exposure (a), BW refers to the average body weight of adults (kg), and AT represents the average time of inhalation exposure (d).

The hazard quotient (HQ) was used to calculate the non-cancer risks (NCR) based on the following equation:

(7) $$TNCR=\sum {HQ}_{i}=\sum \frac{{ADD}_{i}}{{RfD}_{i}}$$

where ${RfD}_{i}$ is the reference dose (mg·m^−3^) of heavy metal i, ${HQ}_{i}$ is the hazard quotient for the non-cancer risk of heavy metal i, and TNCR is the total non-cancer risk. HQ values > 1 indicate that the non-cancer effects are of concern, and HQ values < 1 indicate that the risks could be ignored [33].

The cancer risk of adults is calculated based on the inhalation slope factor (SF, (mg·(kg·day)^−1^)^−1^), and the calculation is based on the following formula:

(8) $$TCR=\sum {CR}_{i}=\sum {ADD}_{i}\;\times \;{SF}_{i}$$

where TCR is the total of ${CR}_{i}$, and ${CR}_{i}$ is the cancer risk of heavy metal i (including Cr, Co, Ni, As, and Cd [34]) for the inhalation pathway. Generally, if ${CR}_{i}\;$< 1 × 10^−6^, the cancer risk is acceptable [33]. The values of EF, ED, AT, BW, RfC, and SF are listed in Table S1.

#### 2.4.2. Source-Specific Health Risk

The source-specific non-cancer and cancer risks were evaluated based on the determined contributions of sources to species from the PMF model [35,36]:

(9) $${HQ}_{k}=\sum_{i}\left({HQ}_{i}\;\times \;{RC}_{ik}\right)$$

(10) $${CR}_{k}=\sum_{i}\left({CR}_{i}\;\times \;{RC}_{ik}\right)$$

where ${HQ}_{k}$ and ${CR}_{k}$ refer to the non-cancer risks and cancer risks induced by the emission source k. ${RC}_{ik}$ is the contribution rate of the specific source to each heavy metal obtained from the source profiles.

Figure S1 provides a detailed scheme of the source-specific health risk assessment approach. Particularly, the concentration of Cr (VI) was assumed to be 1/7 of the total concentration of Cr in the calculation of the health risk [37].

## 3. Results and Discussion

### 3.1. The Mass Concentration and the Characteristics of PM_1_

To present a general discussion of the diurnal and seasonal variations in HMs in PM_1_ near the area of Tianjin International Airport, the temporal variations in PM_1_ and HMs are depicted in Table 1. During sampling, the mean mass concentrations of PM_1_ in the daytime and nighttime were 35.44 ± 16.18 and 30.52 ± 7.49 μg·m^−3^ in spring, and the PM_1_ daily average concentrations were 30.46 ± 14.37 and 23.31 ± 67 μg·m^−3^ in the spring and summer, respectively. The diurnal patterns of PM_1_ in both the spring and summer reveal the phenomenon that the daytime concentration is higher than that at nighttime. Given that there were far more flights during the daytime than at nighttime during our sampling period, the increased aircraft emissions can explain the higher burden of PM_1_ in the daytime in both seasons. However, the seasonality of the PM_1_ concentration in our study showed a relatively inconspicuous variation.

### 3.2. An Overview of the Contamination and PM_1_-Related HMs

#### 3.2.1. HM Contents and Variation

**Figure 2:**
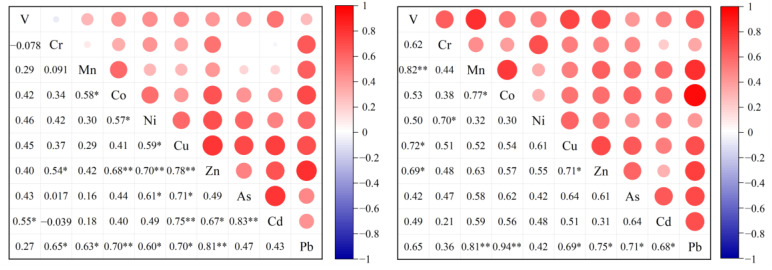
Correlation coefficients and scatter diagrams for HMs of spring (**left**) and summer (**right**) in Tianjin Airport Community. (* and **, correlation coefficients significant at *p* < 0.05 and *p* < 0.01, respectively).

As depicted in Table 1, the sum concentration of ten selected HMs was dominated by Zn and Pb, both in the spring and summer, accounting for a sum concentration greater than 60%. The correlation coefficients for the HMs are exhibited in Figure 2, also showing a correlation between these two HMs. Some studies reported that the high concentration of measured Zn and Pb in the airfield samples could be explained by the aircraft emissions and vehicular tire wear [16,38]. Additionally, the concentrations of Zn and Pb in Tianjin Airport showed similar concentrations to previous studies on other international airports with annual passenger throughputs of millions of people [15,16], but they were higher than the concentrations at the small regional Mytilene airport [39]. This phenomenon indicated that the difference in flight activity density in airports with various scales could affect the concentrations of Zn and Pb. As the priority PM-bound HMs for air pollution control in China, the mean concentrations of As and Cd partly exceeded the corresponding limits of the national ambient air quality standard in China (NAAQS), with values of 17% and 31% among all samples, respectively. After converting the total content of Cr to Cr (VI), the mean concentrations of Cr in our study were all higher than the threshold value [33]. The concentrations of Cr, Mn, Cu, Cd, and Pb reflected a relatively inconspicuous seasonal variation, which could be due to the industrial emissions sources and coal combustion [40].

**Table 1:** Mean concentrations of PM_1_ (μg/m^3^) and HMs (ng/m^3^), background value (BVs, μg/g), and WHO guidelines (yearly basis) about HMs.

	Spring		Summer		BVs ^a^ (μg·g^−1^)	WHO ^b^ (ng·m^−3^)
	Daily (*n*= 7)	Night (*n* = 7)	Daily (*n* = 9)	Night (*n* = 9)		
PM_1_ (μg/m^3^)	35.44 ± 16.18	30.52 ± 7.49	30.46 ± 14.37	23.31 ± 6.67		
V	10.43 ± 5.28	15.65 ± 4.29	3.93 ± 1.90	8.19 ± 3.46	82.4	1000
Cr	7.42 ± 0.68	7.12 ± 0.79	9.79 ± 2.84	8.71 ± 0.86	61.0	-
Mn	25.63 ± 6.26	22.85 ± 9.76	21.68 ± 8.01	20.88 ± 3.94	583.0	150
Co	3.41 ± 0.77	3.63 ± 1.13	1.96 ± 0.49	2.13 ± 0.44	12.7	-
Ni	12.36 ± 3.04	13.58 ± 3.24	9.52 ± 3.43	4.28 ± 1.31	26.9	25
Cu	24.81 ± 13.37	33.17 ± 18.15	26.02 ± 5.77	25.07 ± 6.07	22.6	-
Zn	160.85 ± 22.50	156.38 ± 36.14	161.21 ± 44.31	173.89 ± 26.16	74.2	-
As	4.60 ± 1.49	5.59 ± 1.86	5.63 ± 3.07	5.87 ± 2.12	11.2	6.6
Cd	5.62 ± 1.77	5.83 ± 2.40	7.09 ± 4.49	4.96 ± 1.02	0.097	5
Pb	48.32 ± 15.46	44.39 ± 24.51	55.98 ± 29.92	45.55 ± 15.12	26.0	500

^a^ The background values (BVs, μg·g^−1^) of HMs in Chinese soil, obtained from China National Environmental Monitoring Centre (CNEMC) [25]. ^b^ The corresponding limits of part HMs were regulated by the WHO [41].

#### 3.2.2. The Relationships between the Concentrations of HMs and Flight Activities

**Figure 3:**
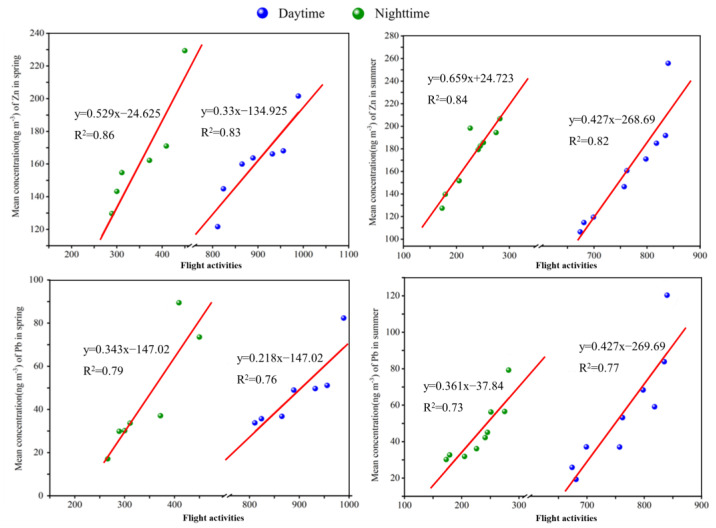
Correlation analysis of flight number and concentrations of Zn and Pb.

To determine the indicator of the aircraft emissions in the airport area, linear regression was used to explore the relationships between flight operations and measured HMs concentrations. Table S2 shows the coefficient of determination $R^{2}$ and the corresponding *p*-values. For Zn and Pb, the variations in their mean concentrations are strongly and linearly correlated with the flight operations during the sampling periods (|$R^{2}$| > 0.7 and p-value < 0.05), and Figure 3 shows the fitting plots between the flight activities and concentrations of Zn and Pb, including the regression slopes, intercepts, and $R^{2}$.

Zn was regarded as a reliable tracer for traffic emissions and was considered based on brake dust, tire wear abrasion, and lubricants in engines [42]. In the airport community, Amato et al. [15] reported that a high concentration of Zn near the airport area was sourced from tire detritus/smoke in runway dust during aircraft landing, and a similar result was obtained by Bennett et al. [43], who collected dust samples from the undercarriage and wheel hubs during landing and braking of aircraft, suggesting that Zn potentially arose from the burning of tire rubber, asphalt tar, or brake abrasion. Furthermore, Gagne et al. [14] suggested that the Zn detected by ADF-STEM from the soot particles in the aviation engine can be associated with lubrication oil. As for Pb, some studies indicated that the Pb measured in the airport can be explained by the aircraft emissions at the airport [38,39]. Turgut et al. [44] analyzed trace elements in collected PM samples of aircraft piston engines under different engine operation states and showed that Pb was the most abundant element among the selected trace elements, which directly indicated that Pb was one of the indicators of aircraft engine exhaust.

Combined with the possible sources of Zn and Pb in the airport area and the strong correlation between flight operations and their variations, it was shown that aircraft activities had a notable impact on the concentrations of Zn and Pb. Thus, Zn and Pb were chosen as the indicators of aircraft emissions in our study. However, due to the lack of data on other aircraft-related emission sources such as special vehicles or power units, i.e., auxiliary power units (APUs) and ground power units (GPUs), in the airport, the impact on the Zn and Pb concentrations in these emissions is not considered in this study.

### 3.3. Seasonal-Specific Source Apportionment Using PMF

The sources of PM_1_-bound HMs in the Tianjin airport area in two seasons were identified based on EF values, a Pearson correlation analysis, and the results of the PMF model. A detailed discussion of the EF values of HMs is presented in Text S2 and Figure S2. Based on the results of the PMF model, four factors were chosen for the source identification of HMs both in the spring and summer. Specifically, the data input in the PMF model was the total from the daytime and nighttime, and the results of source apportionment were based on daily resolution.

#### 3.3.1. Source Apportionment in Spring

**Figure 4:**
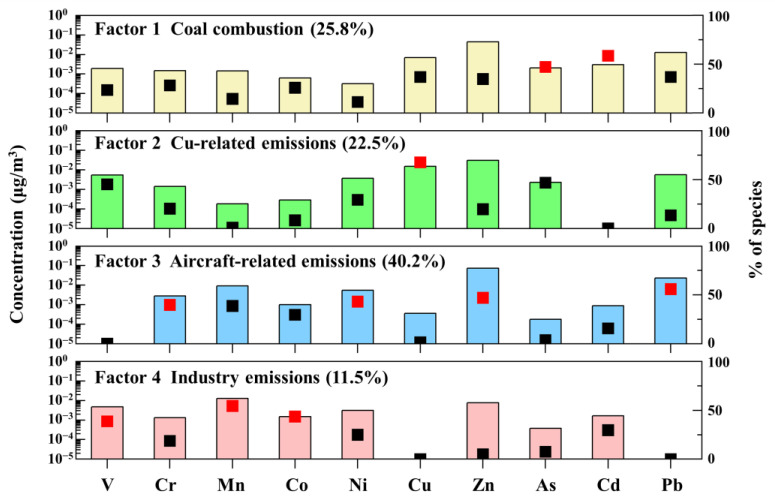
Source profiles of spring obtained using PMF model. Bars represent mass concentrations for pollution sources (yellow column: coal combustion; green column: Cu-related emissions; blue column: aircraft-related emissions; red column: industry emissions) and squares represent contribution percentages from each source factor.

Four potential sources in the spring were confirmed, including coal combustion (25.9%), Cu-related sources (22.4%), aircraft-related sources (40.2%), and industrial sources (11.5%), and the source profiles from the spring are shown in Figure 4.

The first factor was regarded as the coal combustion because of the high contents of Cd (55.6%) and As (42.0%). Cd and As were recognized as tracers of coal combustion in several studies [45]. The EF values of Cd and As showed that they were severely affected by anthropogenic activities both during the daytime and nighttime, and the strong correlation between Cd and As ($r^{2}$ = 0.83, *p* < 0.01) indicated that they might have originated from the same source. As the main industrial emission sources in the airport community, the emissions of coal-fired thermoelectric plants in the Xinli Industrial Park and Baoyuan Industrial Park contributed greatly to coal combustion in the Tian Airport Community.

The second factor was regarded as the anthropogenic source, with high loadings of Cu (67.7%). Cu is an indicator of vehicle emissions, such as automotive lubricants, metal automobile parts, and tire wear [46]. In addition, several studies have indicated that the ground service equipment (GSEs) of aircraft such as carriers, refilling trucks, passenger buses, and contained loaders also contributed to the PM concentration in the airport area [47,48]. Therefore, vehicles serving the aircraft in the airport could also be seen as one of the sources of Cu. Some studies also found that Cu is an important tracer of industrial emissions, pointing to the metal-working, iron, and steel industries [49]. The dominant wind direction in spring is southwest, and there were several metal-processing factories located in the southwest of the sampling site, which could be regarded as the main source of industrial emissions of Cu in the airport community.

The aircraft-related sources consisted of high percentages of Pb (55.9%), Zn (47.0%), Ni (43.1%), and Cr (39.8%). As shown in Figure 2, there are different degrees of correlation between Pb, Zn, Ni, and Cr. Pb and Zn measured in the airport community were chosen as indicators of aircraft emissions. Gagne et al. ^14^ found that Ni is abundant in distillate fuels used in aircraft engines. Moreover, studies also indicated that the landing and take-off processes of aircraft, diesel-powered tugs, and carts could also contribute to a load of Ni in the airport area [38,43,50]. Cr was also found in the PM1 near the airport runway during landing and take-off [16]. As illustrated above, the third source could be regarded as the aircraft-related source.

The source with high loads of Mn (56.9%), Co (47.9%), and V (44.4%) was identified as industrial emissions. In this study, the correlation between Mn and Co is good, and they are considered to be good markers of industrial emissions like metal processing [51]. V mainly presents in emissions from heavy and crude oil combustion or related industries [52,53]. Considering that there were several crude oil processing plants in the two industrial parks near our sampling site, the source of V could be regarded as crude oil-related industries.

#### 3.3.2. Source Apportionment in Summer

**Figure 5:**
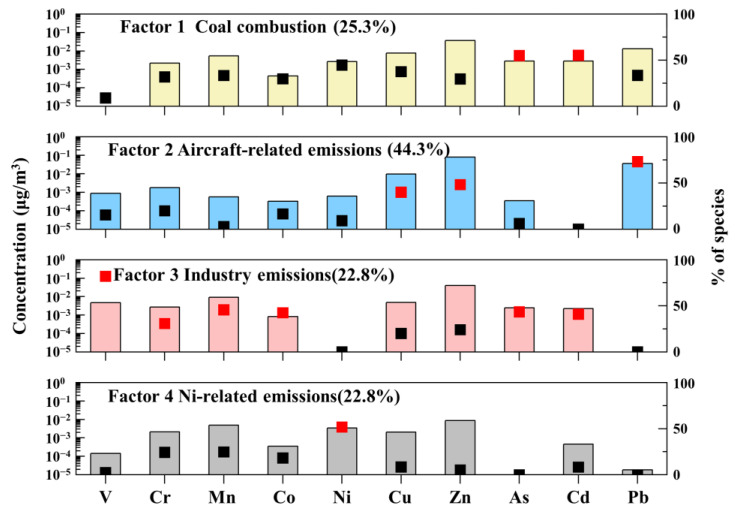
Source profiles of summer obtained by the PMF model. Bars represent mass concentrations for pollution sources (yellow column: coal combustion; blue column: aircraft-related emissions; red column: industry emissions; gray column: Ni-related emissions) and squares represent contribution percentages from each source factor.

As listed in Figure 5, four factors consisting of coal combustion (25.3%), aircraft-related sources (44.3%), industrial sources (40.4%), and Ni-related sources (7.6%) were essential to adequately explain the potential sources of HMs in the summer.

Factor 1 was identified as coal combustion with high percentages of As (55.8%) and Cd (50.4%). Factor 2 was identified as aircraft-related sources characterized by high loads of Pb (73.2%), Zn (48.2%), and Cu (40.1%). As mentioned in Section 3.2.2 and Section 3.3.1 above, Pb and Zn were regarded as indicators of aircraft-related emissions, and vehicles in the airport could also be seen as one of the main sources of Cu. Factor 3 was associated with high contents of V (55.6%), Mn (45.6%), As (43.4), Co (42.4%), Cd (40.9%), and Cr (30.8%). Mn and Co were indicators of industrial activities [51]. V mainly came from emissions due to heavy fuel oil combustion or fuel-oil-related industrial activities [53], and Cr mainly came from industrial emissions such as metal processing [42]. As and Cd have typically been used as tracers of coal combustion [54]. Thus, these sources could be interpreted as industrial sources.

Factor 4 was considered to be Ni-related sources with high loads of Ni (51.7%). Ni was abundant in heavy and crude oil combustion [55]. Ni could also be measured from aircraft exhausts with distillate fuels [14]. However, the EF values of Ni in the summer were non-enriched, indicating that it was hardly affected by anthropogenic activities. Previous studies indicated that Ni is mainly produced by natural weathering and soil-forming parent material [56,57]. Therefore, this factor is associated with Ni sources consisting of natural sources and human activities.

### 3.4. Seasonal Health Risk of HMs

The daily average exposure of HMs in PM_1_ was calculated by using the health risk assessment model, and the cancer and non-cancer risks of HMs to people in the airport community were analyzed based on inhalation exposure (Table 2). For the non-cancer risks of HMs, the TNCRs for all HMs were 0.76 in the spring and 0.63 in the summer, which are lower than the threshold level determined by the USEPA. As the indicators of industrial emissions [58], Mn and Co accounted for more than 90% of the TNCR in both the spring and summer. Furthermore, Mn was the primary contributor to the non-cancer risk among HMs in both seasons, which is similar to a previous study [59].

In summary, the total non-cancer risk of HMs was below 1, indicating that there was no significant non-cancer risk in the Tianjin Airport Community. The comparison of TNCR and TCR through inhalation from the PM-bound HMs is summarized in Table 3. Compared to other studies, the non-cancer risk of HMs in the Tianjin Airport Community was comparable to the results in harbors (Huzhou) and schools [20,60] but much lower than that in some urban areas in China [24,61,62].

The TCR of HMs (Cr, Co, Ni, As, and Cd) was 6.45 × 10^−5^ in the spring and 6.59 × 10^−5^ in the summer (Table 2), which are higher than the safety level [33], implying a potential cancer risk. As shown in Table 3, our cancer risk values had the same order of magnitude as studies on schools (Los Angeles, CA, USA), residential districts (Ulsan, Republic of Korea), and urban areas (Chengdu, China) [63,64,65]. It was found that As creates the highest cancer risk, both in the spring and summer, which was similar to previous studies [60,66], and As has always been regarded as a marker for coal combustion [67]. However, as an indicator of heavy oil [35] and one of the components in aircraft distillate fuels [14], the cancer risks of Ni in the summer were below the acceptable risk level (1 × 10^−6^) [33].

**Table 3:** The comparison of TNCR and TCR through inhalation from the PM-bound HMs.

Study	Study Area	Particle Size	HMs Compositions	TNCR	TCR
Sax [63]	School, Los Angeles, USA	PM_2.5_	Cr, Ni, As, Cd, Pb, and Be	-	1.2 × 10^−5^
	School, New York City, USA				9.6 × 10^−5^
Hieu [64]	Residential district, Ulsan, Republic of Korea	PM_1.0_	Cr, Mn, Ni, Cu, Cd, and Pb	-	9.0 × 10^−5^
Pandey [68]	Residential district, Lucknow, India	PM_2.5_	Fe, Cr, Ni, Cu, Cd, and Pb	-	1.1 × 10^−5^
Zhang [61]	Urban area, Taiyuan, China	PM_2.5_	Cr, Mn, Co, Ni, Cu, Zn, Cd, and Pb	6	-
Peng [20]	Harbor, Huzhou, China	PM_2.5_	Cr, Co, Ni, As, Cd, and Pb	0.15	1.5 × 10^−4^
Zhou [69]	Urban area, Pearl River Delta region, China	PM_2.5_	Cr, Ni, Mn, As, and Pb	2.09	3.37 × 10^−4^
Liu [60]	School, Beijing, China	PM_2.5_	Ba, V, Cr, Mn, Co, Ni, As, Cd, and Pb	0.89	7.04 × 10^−6^
Huang [62]	Urban area, Shanghai, China	PM_2.5_ and PM_10_	Mn, Cu, Zn, and Pb	2.99 (PM_2.5_) and 3.18 (PM_10_)	-
Huang [70]	School, Beijing, China	PM_2.5_	Ti, V, Cr, Mn, Co, Ni, As, Cd, and Pb	1.15	1.14 × 10^−4^
Xie [35]	Urban area, Pearl River Delta and Yangtze River Delta, China	PM_2.5_	Fe, V, Cr, Mn, Co, Ni, Zn, As, Cd, and Pb	-	0.68–1.3 × 10^−5^ (PRD) and 1.9–2.7 × 10^−5^ (YRD)
Cui [66]	Urban area, Beijing, China	PM_2.5_	Ba, Cr, Mn, Ni, As, and Pb	>100	1.1 × 10^−2^
Xu [24]	Urban area, Zhejiang Province, China	PM_2.5_	Cr, Mn, Co, Ni, As, Cd, and Pb	11.9	1.7 × 10^−3^
Chen [59]	School, Tianjin, China	PM_2.5_	Cr, Mn, Co, Ni, Cu, Zn, As, and Pb	1.3	2.76 × 10^−5^
Sun [65]	Urban area, Chengdu, China	PM_10_	V, Cr, Mn, Co, Ni, Cu Zn, As, Cd, and Pb	2.9–6.9	8.2–11 × 10^−5^

### 3.5. Source-Specific Assessment of Health Risk

**Figure 6:**
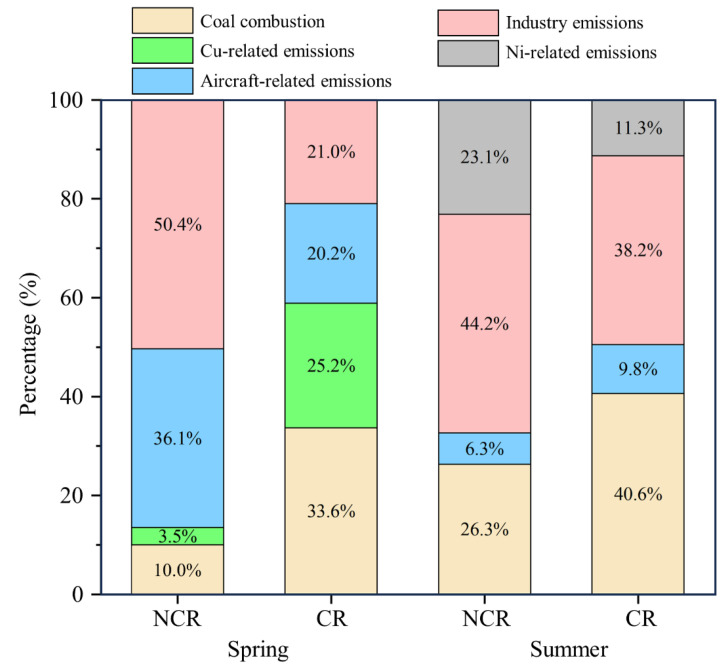
The source-specific health risk and their quantifications in the spring and summer.

In order to protect human health, a source-specific risk assessment is an effective tool for prioritizing heavy metal sources in environmental control [71]. Therefore, based on the mass contributions of samples from the PMF profiles, the non-cancer and cancer risk values from different sources in the Tianjin Airport Community were calculated. The source-specific health risk and their quantification in the spring and summer are shown in Figure 6.

For the non-cancer risk, the contribution of each source of non-cancer risk values was ranked as follows (values of HQ and CR were shown in Table S3): industrial sources (50.4%) > aircraft-related sources (36.1%) > coal combustion (10.0%) > Cu-related sources (3.5%) in the spring; industrial sources (44.2%) > coal combustion (26.3%) > Ni-related sources (23.1%) > aircraft-related sources (6.3%) in the summer. Industrial sources were the predominant contributor in both seasons, and the primary contribution of Mn to the non-cancer risk could account for why industrial sources contributed the most to the TNCR values in two seasons, similar to a previous study [36]. These results suggest that industrial sources should be considered as the priority pollution source for the Tianjin Airport Community.

For the cancer risk, the values of individual sources in both the spring and summer were all higher than the acceptable level (1 × 10^−6^). Among all of the factors, coal combustion was the major sources in both seasons, and their cancer risk values were 21.70 × 10^−6^ in the spring and 26.76 × 10^−6^ in the summer, accounting for 33.6% and 40.6% of the total risk, respectively. As and Cd were indicators of coal combustion and occupied the largest proportion of the cancer risk of coal combustion, meaning this factor ranked at the top of TCR, similar to the results of previous studies [59,60]. Therefore, coal combustion-related activities in several industrial parks need to be prioritized in the Tianjin Airport Community. Although the source contributions of aircraft-related emissions based on the PMF profiles were the largest among all sources in both seasons, the CR of aircraft-related sources was much lower than those of the other sources.

## 4. Conclusions

To investigate the seasonal and diurnal variations in the concentrations, sources, and source-specific health risks of PM_1_-bound HMs in the airport community, samples of PM_1_ in the Tianjin Binhai International Airport Community were collected. The relatively high intensity of aircraft activities during the daytime revealed that the PM_1_ diurnal variation in the concentration in the daytime was higher than that at nighttime both in the spring and summer. Zn and Pb were regarded as indicators of aircraft emissions in this study. After comprehensive source apportionment with EF values, a correlation analysis, and the PMF model, four factors including coal combustion, Cu-related sources, aircraft-related sources, and industrial sources were determined in the spring, and four factors including coal combustion, aircraft-related sources, industrial sources, and Ni-related sources were identified in summer.

The TNCR values in the spring and summer were lower than the limit values. On the other hand, the TCR values of carcinogenic HMs in the spring and summer both exceeded the limit, and As posed the largest cancer risk. Additionally, the source-specific results show that the industrial sources related to Mn were the dominant sources for the non-cancer risk, making the proportion 50.4% in the spring and 44.2% in the summer, respectively. Coal combustion with an abundance of As was the leading source of cancer risk instead of aircraft-related sources and contributed the most to the concentration of HMs among source contributions. To obtain a better understanding of the health effects of PM_1_-bound HMs, more attention should be paid to source-specific health risks and targeted efforts should be adopted to control the management of industry sources and coal combustion for public health in the Tianjin Airport Community instead of focusing on aircraft-related emissions. However, as we were limited by sampling conditions, the emission inventory around the airport community was not determined, and we were unable to verify the results of source appointment. Taken together, these results could provide essential information for assessing the impact of airport-community sources on HM emissions in the receptor environment and are therefore promising for air pollution monitoring and control.

## Figures and Tables

**Table 2:** Non-cancer and cancer risks for each PM_1_-bound HM in spring and summer.

HMs	Non-Cancer Risk $({HQ}_{inh}$)		Cancer Risk $({CR}_{i}$)	
	Spring	Summer	Spring	Summer
V	5.96 × 10^−4^	2.78 × 10^−4^	-	-
Cr (VI)	1.16 × 10^−2^	1.48 × 10^−2^	1.39 × 10^−5^	1.77 × 10^−5^
Mn	5.42 × 10^−1^	4.76 × 10^−1^	-	-
Co	1.97 × 10^−1^	1.14 × 10^−1^	1.10 × 10^−5^	6.40 × 10^−6^
Ni	2.01 × 10^−4^	1.07 × 10^−4^	3.48 × 10^−6^	1.85 × 10^−6^
Cu	2.31 × 10^−4^	2.03 × 10^−4^	-	-
Zn	1.69 × 10^−4^	1.79 × 10^−4^	-	-
As	5.42 × 10^−3^	6.10 × 10^−3^	2.46 × 10^−5^	2.78 × 10^−5^
Cd	1.83 × 10^−3^	1.93 × 10^−3^	1.15 × 10^−5^	1.21 × 10^−5^
Pb	4.22 × 10^−3^	4.63 × 10^−3^	-	-
TNCR/TCR	7.62 × 10^−1^	6.26 × 10^−1^	6.45 × 10^−5^	6.59 × 10^−5^

## Data Availability

The data presented in this study are available on request from the corresponding author.

## Supplementary Materials

The following supporting information can be downloaded at: https://www.mdpi.com/article/10.3390/toxics12080601/s1, Text S1: Chemical analysis and quality control of heavy metals; Text S2: The enrichment factor (EF) values of HMs [21,53,72,73]; Figure S1: Schema of the source-specific health risk assessment in the Tianjin Airport community; Figure S2: The EFs values of HMs in spring and summer; Table S1: Parameters and their detailed description for the health risk assessment [18,33,34,36]; Table S2: Values of the R2 of flight data and HMs concentrations; Table S3: Source contribution of sources and the source-specific health risk in spring and summer.
