# Supplementary material for: Pollution Characteristics of Heavy Metals in PM1 and Source-Specific Health Risks in the Tianjin Airport Community, China
Source: Toxics. 2024 Aug 18;12(8):601. doi: 10.3390/toxics12080601 (PMC11359593; doi:10.3390/toxics12080601)
Supplement: Supplementary file 1 [file toxics-12-00601-s001.zip › toxics-3122078-supplementary.pdf]

Supporting Information for:

Pollution Characteristics of Heavy Metals in PM_1 and Source-Specific Health Risks in the Tianjin Airport Community, China

Jingbo Zhao [1], Jingcheng Xu [1], Yanhong Xu [1] and Yaqin Ji [2,*]

[1] College of Transportation Science and Engineering, Civil Aviation University of China, Tianjin 300300, China
[2] College of Environmental Science and Engineering, Nankai University, Tianjin 300350, China
* Correspondence: jiyaqin@nankai.edu.cn

Submitted to

Number of pages: 7

Number of texts: 2

Number of figures: 2

Number of tables: 3

Contents of this file

Text S1 Chemical analysis and quality control of heavy metals

Text S2 The enrichment factor (EF) values of HMs

Figure S1 Schema of the source-specific health risk assessment in the Tianjin Airport community

Figure S2 The EFs values of HMs in spring and summer

Table S1 Parameters and their detailed description for the health risk assessment.

Table S2 Values of the R^2 of flight data and HMs concentrations

Table S3 Source contribution of sources and the source-specific health risk in spring and summer

Reference

Text S1 Chemical analysis and quality control of heavy metals

A quarter of the filter was cut using acid-cleaned ceramic scissors and then extracted by a mixture of concentrated nitric acid (65% GR grade; 6 mL) and hydrochloric acid (37% GR grade; 2 mL) in a microwave digestion system (MARS 5, CEM CO., USA). The temperature program for digestion was set as follows: ramp from the room temperature to 185 ℃ in 15 min and hold for 25 min for complete digestion. After cooling down to room temperature, the extract was filtrated through a 0.45 mm pore size membrane. The vial was rinsed twice with Milli-Q water, and the rinsing solution was also filtrated the same way as the extract. The collected extract was further diluted to 50 mL, and this final extract was stored in a metal-free polyethylene vial at 4℃ until analysis. The extract was analyzed on an inductively coupled plasma e mass spectrometry (ICP-MS, Agilent 7500a) for 10 heavy metals, namely V, Cr, Mn, Co, Ni, Cu, Zn, As, Cd and Pb. External calibrations were obtained from authentic standards (Sigma-Aldrich, St. Louis, MO, USA). All analytical results were corrected by subtracting values from blank filters, which were determined the same way as actual samples. Daily

optimization was performed on the instrument according to the standard protocols and criteria before sample analysis. The average repeatability of the target trace elements was <7%, and the recoveries ranged from 91.2% to 106.7%, determined by spiking a certified standard mix on acid-washed blank filters.

Text S2 The enrichment factor (EF) values of HMs

The EF values for HMs in the PM_1 were calculated following Eq. (S1) and were classified into three groups (Fig. S2): (A) Co and Ni. These HMs were mostly not enriched with EFs < 10, suggesting that they were rarely affected by human activities or primarily from crustal origins such as soil dust[1]. (B) Cr, V, Cu, Pb, Zn, and As. These metals were moderately enriched(10 < EFs < 100), suggesting the dominance of anthropogenic sources[2]. (C) The EF of Cd was >100, indicating its abundance was highly enriched by anthropogenic activities, such as heavy fuel combustion, coal burning, and road traffic emissions[3].

The average calculated EF values for 10 selected HMs varied widely from 4.1 to 1967, showing similar diversity to other reports[4]. It is worth noting that the diurnal variation of EFs in spring presented the feature that the EF values in daytime were almost higher than that at nighttime, indicating that there were more serious pollution levels during the daytime, while the feature was insignificant in summer. However, EF values of individual HM did not display strong seasonal variations and they were all of the same magnitudes.

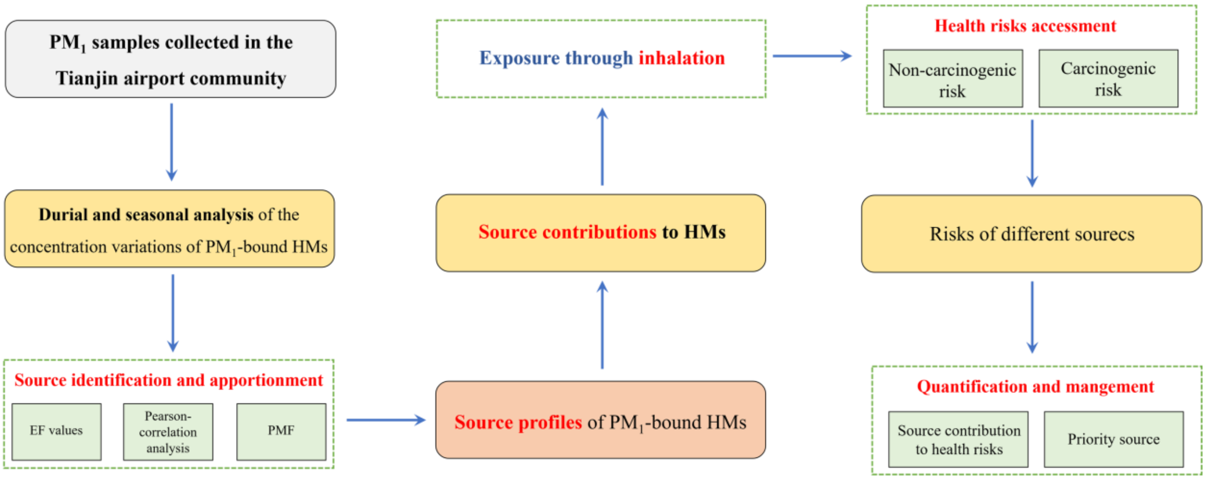

Figure S1 Scheme of the source-specific health risk assessment in the Tianjin Airport community.

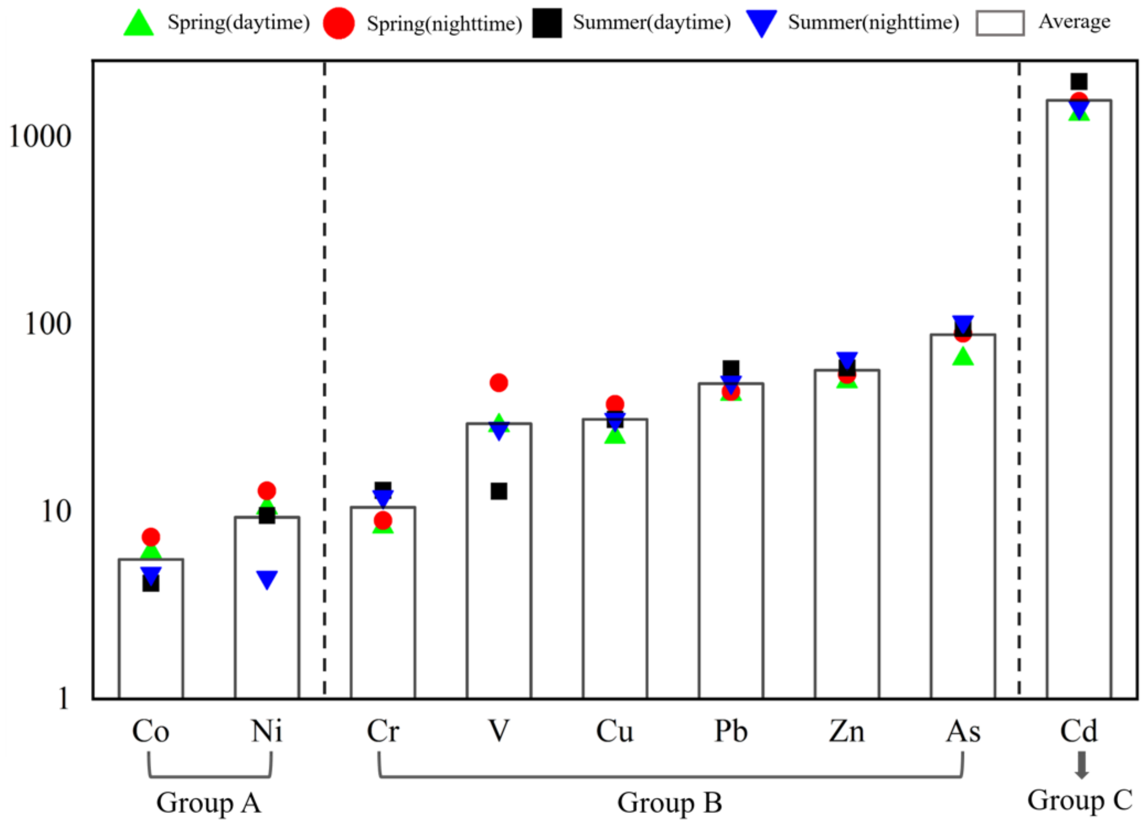

Figure S2 The EFs values of HMs in spring and summer.

Table S1 Parameters used in the calculation of carcinogenic risks and hazard quotients in this study.

Parameters	Unit	Values (adults)	HMs	RfD^a (mg kg^{-1} day^{-1})	SF^b ((mg kg^{-1} day^{-1})$^{-1}$)
$InhR^c$	m^3/d	20	V	7.00×10^{-3}	-
ED^c	a	30	Cr	2.86×10^{-5}	42.0
EF^c	d/a	350	Mn	1.43×10^{-5}	-
AT^d	d	30×365	Co	5.71×10^{-6}	9.80
BW^d	kg	60	Ni	2.06×10^{-2}	8.40×10^{-1}
			Cu	4.02×10^{-2}	-
			Zn	3.00×10^{-1}	-
			As	3.01×10^{-4}	15.1
			Cd	1.00×10^{-2}	6.3
			Pb	3.50×10^{-3}	-

a The values of RfD obtained from Chen, et al. [5];

b SF obtained from USEPA [6];

c $InhR$, ED, and EF obtained from USEPA [7];

d AT and BW values obtained from Zhang et al.[8];

Table S2 Values of the R^2 of flight data and HMs concentrations.

	Spring				Summer			
	Daytime		Nighttime		Daytime		Nighttime	
	R^2	P	R^2	P	R^2	P	R^2	P
V	0.41	0.123	0.36	0.161	0.18	0.248	0.53*	0.011
Cr	0.23	0.964	0.54	0.061	0.42	0.060	0.46	0.106
Mn	0.63*	0.033	0.66	0.328	0.29	0.103	0.57*	0.020
Co	0.54	0.060	0.30	0.203	0.23	0.196	0.50*	0.033
Ni	0.48	0.085	0.60*	0.041	0.46	0.045	0.43	0.055
Cu	0.66*	0.027	0.82**	0.005	0.49*	0.019	0.62*	0.011
Zn	0.83**	0.004	0.87	0.002	0.85**	0.000	0.85**	0.000

As	0.37	0.147	0.45	0.098	0.62*	0.011	0.54*	0.024
Cd	0.60*	0.041	0.45	0.100	0.65**	0.009	0.58	0.071
Pb	0.76**	0.010	0.79**	0.007	0.77**	0.002	0.73**	0.002

* indicated that $p < 0.05$ while ** indicated that $p < 0.01$

Table S3 Source contribution of sources and the source-specific health risk through inhalation in spring and summer.

	Source	Source contribution (%)	HQ	CR ($\times 10^{-6}$)
Spring	Coal combustion	25.8	0.08	21.70
	Cu-related emissions	22.5	0.03	16.28
	Aircraft-related emissions	40.2	0.27	13.01
	Industry emissions	11.5	0.39	13.53
	Subtotal	100	0.76	64.52
Summer	Coal combustion	25.3	0.16	26.76
	Aircraft-related emissions	44.3	0.03	6.48
	Industry emissions	22.8	0.27	25.18
	Ni-related emissions	7.6	0.14	7.43
	Subtotal	100	0.63	65.88

Reference

1. Xue, Q.; Jiang, Z.; Wang, X.; Song, D.; Huang, F.; Tian, Y.; Huang-fu, Y.; Feng, Y., Comparative study of PM10-bound heavy metals and PAHs during six years in a Chinese megacity: Compositions, sources, and source-specific risks. *Ecotoxicology and Environmental Safety* **2019,** *186*, 109740.

2. Nriagu, J. O.; Pacyna, J. M., Quantitative assessment of worldwide contamination of air, water and soils by trace metals. *Nature* **1988,** *333*, (6169), 134-139.

3. Deng, S.; Shi, Y.; Liu, Y.; Zhang, C.; Wang, X.; Cao, Q.; Li, S.; Zhang, F., Emission characteristics of Cd, Pb and Mn from coal combustion: Field study at coal-fired power plants in China. *Fuel Processing Technology* **2014,** *126*, 469-475.

4. Li, H.; Wang, Q. g.; Yang, M.; Li, F.; Wang, J.; Sun, Y.; Wang, C.; Wu, H.; Qian, X., Chemical characterization and source apportionment of PM2.5 aerosols in a megacity of Southeast China. *Atmospheric Research* **2016,** *181*, 288-299.
5. Chen, R.; Zhao, Y. H.; Tian, Y. Z.; Feng, X.; Feng, Y. C., Sources and uncertainties of health risks for PM2.5-bound heavy metals based on synchronous online and offline filter-based measurements in a Chinese megacity. *Environment International* **2022,** *164*.
6. Huang, J.; Guo, S.; Zeng, G.-m.; Li, F.; Gu, Y.; Shi, Y.; Shi, L.; Liu, W.; Peng, S., A new exploration of health risk assessment quantification from sources of soil heavy metals under different land use. Environmental Pollution 2018, 243, 49-58.
7. Tepanosyan, G.; Sahakyan, L.; Belyaeva, O.; Maghakyan, N.; Saghatelyan, A., Human health risk assessment and riskiest heavy metal origin identification in urban soils of Yerevan, Armenia. Chemosphere 2017, 184, 1230-1240.
8. Zhang, Q.; Yang, L.; Peng, J.; Wu, L.; Mao, H., Characteristics, sources, and health risks of inorganic elements in PM2.5 and PM10 at Tianjin Binhai international airport. *Environmental Pollution* **2023,** *332*, 121988.
